# The development and implementation of a 12-month simulation-based learning curriculum for pediatric emergency medicine fellows utilizing debriefing with good judgment and rapid cycle deliberate practice

**DOI:** 10.1186/s12909-018-1417-6

**Published:** 2019-01-15

**Authors:** Justin M. Jeffers, Shannon Poling

**Affiliations:** 1 Department of Pediatrics, Bloomberg Children’s Center, Division of Pediatric Emergency Medicine, Johns Hopkins University School of Medicine, Suite G-1509, 1800 Orleans St, Baltimore, MD 21287 USA; 2 Johns Hopkins Medical Simulation Center, Johns Hopkins University, 600 North Wolfe Street, Blalock 701, Office 702A, Baltimore, MD 21287 USA

**Keywords:** Curriculum development, Medical simulation, Pediatric emergency medicine training, Medical education, Rapid cycle deliberate practice, Debriefing with good judgment, Instructional design

## Abstract

**Background:**

There are currently training gaps, primarily procedural and teamwork skills, for pediatric emergency medicine (PEM) fellows. Simulation-based learning (SBL) has been suggested as an educational modality to help fill those gaps. However, there is little evidence suggesting how to do so. The objective of this project is to develop and implement an SBL curriculum for PEM fellows with established curriculum development processes and instructional design strategies to improve PEM fellowship training.

**Methods:**

We developed a 12-month longitudinal SBL curriculum focused on needs assessment, instructional strategies, and evaluation. The curriculum development process led us to combine the instructional strategies of debriefing with good judgment, rapid cycle deliberate practice, and task-training to improve core PEM skills such as procedural competence, crisis resource management, and managing complex medical and traumatic emergencies. Using multiple approaches, we measured outcomes related to learners (attendance, performance, critical procedure opportunities), instructor performance, and program structure.

**Results:**

Eight/Eight (100%) PEM fellows participated in this curriculum from July 2015 to June 2017 with an overall attendance rate of 68%. Learners self-reported high satisfaction (4.4/5, SD = 0.5) and perceived educational value (4.9/5, SD = 0.38) with the curriculum and overall program structure. Learners had numerous opportunities to practice critical procedures such as airway management (20 opportunities), defibrillator use (ten opportunities), and others (ten opportunities). Learner Debriefing Assessment for Simulation in Healthcare (short version) scores had mean scores greater than 5.8/7 (SD = 0.89) across all six elements.

**Conclusions:**

This longitudinal SBL curriculum combining debriefing with good judgment and rapid cycle deliberate practice can be a feasible method of reducing current training gaps (specifically with critical procedure opportunities) in PEM fellowship training. More work is needed to quantify the training gap reduction and to refine the curriculum.

## Background

Clinical exposure is insufficient for pediatric emergency medicine (PEM) fellows to obtain key procedural and teamwork skills [1–9]. A review in 2013 found that out of 261 critical procedures performed during 194 patient resuscitations, pediatric emergency medicine fellows performed a median of three in a 12-month period [1] . Yet these critical procedures and team-based skills are important and required by the Accreditation Council for Graduate Medical Education (ACGME) [10]. Additionally, this lack of critical procedure exposure has a potential impact on faculty skill retention and therefore, potential patient care [2, 5].

The American Board of Pediatrics (ABP) and the Accreditation Council for Graduate Medical Education (ACGME) through their developmental milestone and entrustable professional activities (EPA) documents have suggested simulation-based learning (SBL) as a modality to help fill the training gap PEM fellows currently experience [11]. This is supported by the growing collection of evidence supporting SBL as an educational modality to improve the care we provide to the sickest patient populations [12–23].

The published literature identifies numerous individual scenarios and one-day curriculums for this learner group but there are no well-developed and well-described longitudinal SBL curriculums for pediatric emergency medicine fellows [24–29]. Prior to this curriculum, PEM fellows at this institution (tertiary care children’s center) did not participate in any formal SBL training.

Currently, there is little evidence guiding choice of instructional strategies (IS) within SBL [30]. A goal during development of this 12-month longitudinal curriculum was to thoughtfully choose IS based on needs assessments, learner objectives, and instructional design principles.

Our overall aim was to develop an SBL curriculum based on instructional design principles to fill training gaps in PEM fellowship. In this article, we describe the design, implementation, and evaluation of this formative assessment curriculum.

## Methods

### Overall curriculum development strategy

The PEM fellowship at this institution is three years and has two fellows per year for a total of six learners per academic year. We used Thomas et al’s 6-step approach to curriculum development [31] in conjunction with instructional design principles, including the Analysis, Design, Development, Implementation, and Evaluation (ADDIE) approach [32] to design this curriculum.

Instructional design is the systematic and reflective process of translating principles of learning into instruction [33]. There are numerous advantages to using systematic instructional design. Most relevant to this learner group include: 1. Encourages advocacy of the learner. 2. Supports effective, efficient, and appealing instruction. 3. Facilitates congruence among objectives, activities, and assessment [33] .

Similar to the way Thomas, et al. provide a systematic approach to curriculum development, the ADDIE approach provides a framework for a systematic approach to the analysis of learning needs, the design and development of a curriculum, and evaluation [34]. The ADDIE approach has been used to improve patient safety, procedural competency, as well as effectively changing clinical practice behaviors [35–37].

Based on these instructional design principles and in an effort to be efficient with the design and delivery of instruction, the curriculum development process for this project condensed the six steps into three: 1. Needs assessment (combining problem identification, needs assessments, and goals and objectives) 2. IS and 3. Evaluation (combining implementation and evaluation).

We implemented our curriculum from July 2015 through June 2017.

### Needs assessment

We conducted four needs assessments to identify core areas in need of improvement and to guide development of our curriculum: [1] A literature search to determine training gaps. [2] A review of ACGME and AAP milestones and EPA’s specific to PEM to ensure fellowship training compliance. [3] An anonymous on-line needs assessment survey of learners prior to participation in the curriculum. [4] A review of the past three years of PEM fellow in-service exam scores to identify low performing areas.

The search phrases, “pediatric emergency medicine training”, “pediatric emergency medicine competencies”, “pediatric emergency medicine education”, “pediatric emergency medicine critical procedures”, “pediatric skill acquisition”, “pediatric skill retention”, “pediatric crisis resource management education” were used to search literature identifying training gaps. The anonymous on-line survey addressed specific learner needs relating to perceived gaps in training and clinical experience.

The needs assessments were synthesized and reviewed to determine core deficits to be addressed. The needs assessments were also combined with the ACGME competencies to develop curricular goals.

### Instructional strategies

Based on the results of the needs assessments, a systematic approach utilizing the ADDIE process was applied to determine the IS to be used [32].

### Evaluation

Attendance was tracked, and all sessions were recorded to promote accurate assessment. The curriculum was evaluated formatively via end of session electronic surveys regarding session objectives, and summatively by an end of curriculum focus group of the six participants lead by the authors and end of curriculum anonymous on-line survey. Multiple assessment tools were used to evaluate and provide feedback to the learners for procedural skills and crisis resource management (CRM) [12, 38–40]. Critical procedure opportunities were tracked via video review. Instructor feedback and assessment was done via the Debriefing Assessment for Simulation in Healthcare Student Version© tool (DASH-SV) [41]. Descriptive analysis was used.

This study was reviewed and approved by our Institutional Review Board. All participants gave written informed to participate in this study. The datasets used and/or analyzed during the current study are available from the corresponding author on reasonable request.

## Results

### ADDIE process

#### Analysis/needs assessments

**Fig. 1:**
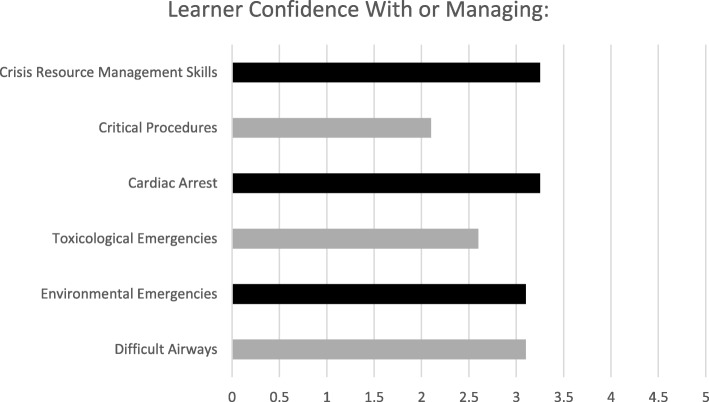
Learner needs assessment: 5-point Likert scale

The literature search identified critical procedures and critical care management items [1, 2, 4, 5], which matched with PEM specific EPA’s. All eight participants completed the learner needs assessment survey prior to participating in the curriculum. Several recurring needs such as procedural, CRM skills, and medical/trauma management of core PEM processes were discovered (Sample shown in Fig. 1). Open-ended survey questions, in-service exam scores of the eight participants and the informal discussions did not add additional needs. The analysis process focused the curriculum into two general categories: [1] Procedural/task skills and [2] CRM/teamwork skills, with the goal of integrating both categories as often as possible.

Table 1 illustrates the competency-based curricular goals in detail. Using the Pediatric Milestone Project and the needs assessments, a curricular goal was written for each of the seven competencies: patient care, medical knowledge, practice-based learning and improvement, interpersonal and communication skills, professionalism, systems-based practice, and personal and professional development. Each goal was linked with an evaluation strategy.

**Table 1:** Global Competency-Based Curriculum Goals with Assessment Strategy

Competency Based Goal (from Pediatric Milestone Project)	By the end of this curriculum, the learner will be able to:
Patient Care	Be proficient at diagnosis and treating a variety of pediatric emergency medicine illnesses and processes using simulation-based learning and apply it to real patients as measured via checklist and direct observation.
Medical Knowledge	Competently recognize and manage pediatric emergencies as well as demonstrate pediatric emergency skills such as high-quality CPR, defibrillation, and other minor procedures as measured via checklist.
Practice-based Learning and Improvement	Critically reflect on their own abilities and generalize to other areas of patient care as measured via structured debriefing and faculty discussions.
Interpersonal and communication skills	Utilize appropriate crisis resource management and communication strategies to improve teamwork as measured via checklist.
Professionalism	Demonstrate patient related professionalism such as empathy as well as career professionalism such as timeliness and interprofessional respect as measured via attendance tracking.
Systems-Based Practice	Integrate into an interprofessional setting and be competent leading a crisis situation regardless of resources as measured via checklist.
Personal and Professional Development	Perform self-directed learning to continue to improve the skills required to be a PEM physician as measured via tracking of articles distributed by learners.

#### Design

Once the needs assessments and goals were completed and synthesized, the study team met to determine IS for the curriculum. After reviewing the literature, we determined that rapid cycle deliberate practice (RCDP) [12, 14] and debriefing with good judgment [42, 43] would best serve our curricular needs and goals.

Rapid Cycle Deliberate Practice is an emerging instructional strategy within SBL that has been shown to be beneficial for high stakes events and procedures such as high quality cardiopulmonary resuscitation, defibrillation, airway management, and team skills [12, 14, 27, 44]. Rapid cycle deliberate practice utilizes a facilitator-guided within-event debriefing approach [45] that maximizes the time learners spend in deliberate practice giving multiple opportunities to practice skills the correct way [14].

Debriefing with good judgement is a well-established debriefing method that uses debriefer observations of performance gaps to explore learner frames and thought processes [42, 43]. It uses a 3-phase (reaction, analysis, and summary) conversational approach based in reflective practice [43, 45]. It can be used to improve a variety of skills such as teamwork and medical management principles.

There are numerous other reflective practice debriefing methods that have been shown to be effective [46–49]. There is currently no definitive literature describing which method is better or best for a given situation or learner objective. The authors chose debriefing with good judgment based on its established track record, its associated assessment tool [50] and author familiarity and comfort. All sessions would be held in the institution’s Medical Simulation Center.

#### Development

The development phase allowed the curriculum calendar to be finalized. A 12-month duration for the curriculum was chosen based on an estimated 66% attendance rate, allowing learners to be exposed to the curriculum twice during their three-year fellowship. This anticipated attendance rate is due to schedule challenges such as off campus rotations, on campus rotations that do not allow learners to participate, work hour rules, as well as past fellowship training conference attendance.

The sessions occurred monthly for 2–4 h (Nine 2-h sessions and three 4-h sessions integrating specific procedural skills) (Table 2). Seasonality was considered as well as the importance of certain core skills. For example, hypothermia from cold water drowning was scheduled for winter and the first cardiac session focusing on pediatric advanced life support and CRM skills occurred early in the academic year.

**Table 2:** Curricular Content Calendar

Month (Hours) Bold Identifies Procedural Skills Days	Content (Core – Specific cases)
July [2]	Cardiac 1 - PEA, VF
August [4]	Trauma - Abdomen, Head
	Skills - Chest Tube, Pericardiocentesis
September [2]	Shock 1 - Septic, Neurogenic
October [4]	Respiratory 1 - Status Asthmaticus, Acute Chest
	Airway Skills Day – Laryngeal Mask Airway, Cricothyrotomy, Difficult Airway Management
November [2]	Toxicology – Tri-Cyclic Antidepressant, Iron
December [2]	Environmental - Drowning (hypothermia), Electrocution
January [2]	Cardiac 2 – Supraventricular Tachycardia, Pulseless Ventricular Tachycardia
February [2]	Respiratory 2 - Upper Airway Obstruction, Aspiration pneumonia
March [2]	Endocrine – Diabetic Ketoacidosis with Cerebral Edema, Thyroid Storm
April [2]	Renal - Hypertensive Emergency, Acute renal failure leading to ventricular fibrillation
May [2]	Oncology - Mediastinal Mass, Hyperleukocytosis
June [4]	Shock 2 - Cardiogenic, Hypovolemic Access Skills

Every month had a general core concept or need to be addressed such as cardiac or toxicology emergencies. Each two-hour session had two scenarios. One utilized debriefing with good judgment to focus on mental models, teamwork, and thought processing to fill in performance gaps. To promote retention and generalization, the second scenario was often related to the first. It utilized RCDP to focus on procedural skills, muscle memory, and integration of multiple ongoing processes such as advanced airway management concurrent with high quality cardiopulmonary resuscitation. The four-hour sessions included procedural skills such as ultrasound and advanced airway management. These skills were integrated into scheduled scenarios for that session. A typical 4-h procedural session consisted of a 2-h skills workshop followed by two scenarios to provide clinical context and reinforce the recently acquired skills. Procedural, teamwork, and CRM skills were reinforced numerous times throughout the curriculum to promote retention. For example, airway management procedures were needed during numerous months and not just during the airway skills session.

The purpose of focusing on monthly core concepts was to allow some flexibility in scenario usage. Specifically, flexibility in relating to recent real patients allowing for more reinforcement and generalization. For example, if the core concept was abdominal trauma and there was a recent real abdominal trauma event that presented a challenge, a scenario would be written to reflect that real-life situation as opposed to a generic scenario.

The primary author was the core educator and was present at all sessions. The second author, a medical simulation center medical educator and respiratory therapist, was present for most sessions and offered expertise with the development and content delivery of the curriculum. Other content experts were invited to participate when appropriate. For example, a PEM ultrasound expert participated during ultrasound heavy sessions.

#### Implementation

For three months prior to the July 2015 full implementation, core concepts were piloted. Minor adjustments were made to those scenarios related to time management. One four-hour skills day (access) was piloted during that time. No adjustments were required. This pilot period provided an outline for the development of scenarios and skills day structure throughout the curriculum. The pilot period also supported the estimated 66% attendance rate.

#### Evaluation/feedback

Evaluation strategies focused on five areas: [1] Attendance. [2] Learner satisfaction, perceived educational value, and potential curricular changes from end of curriculum survey and focus group session. [3] Specific scenario objectives from end of session survey. [4] Number of critical procedure opportunities per observation and video review. [5] Instructor performance per learner completed DASH-SV© forms. A sixth area, quantitative performances on certain critical procedural skills via various checklists and assessment tools is not reported due to low *n* but is ongoing.

Attendance for the year-long curriculum was 68% (65/96 possible participation opportunities). Given the low *n* of eight, quantitative performance assessments have been collected but not analyzed. Overall, the learners found self-reported high satisfaction (4.4/5, SD = 0.5) and educational value (4.9/5, SD = 0.38) in the curriculum with an 88% response rate (7/8 participants) but did suggest areas of improvement and future direction (Table 3). The end of curriculum focus group further reinforced these future changes. End of session electronic surveys asking if objectives were met for each learning session had a score of 4.7/5 (SD = 0.62) with 82% survey completion (53/65).

**Table 3:** Future Directions

Change	Implementation
Increase Ultrasound Training	Review curriculum and discuss with ultrasound expert regarding more ways to integrate ultrasound training and practice
Increase Realism	Move some months to an in-situ setting and recruit pediatric emergency department nurses, technicians, faculty, and other learners such as emergency medicine and pediatric residents
Include Medical Education Component	Create an adjunct curriculum focusing on adult learning, basic SBL skills, and SBL related instructional strategies leading to opportunities for the learners to participate in a peer-to-peer SBL educational process

Learners were exposed to numerous critical procedure opportunities (often multiple opportunities per session) during the 12-month curriculum. Each learner had up to 20 airway skills opportunities, ten defibrillator use opportunities, and ten opportunities for other procedures such as pericardiocentesis, central line insertion, and chest tube insertion. Mean total critical care procedures performed were 27 (SD = 2.4), 67.5% of total opportunities.

Learner DASH-SV© evaluations were done quarterly for a total of 21 evaluations (consistent with attendance rates). Mean scores for all six elements were above 5.8 (maximum of seven). The lowest scoring element was element three (The instructor structured the debriefing in an organized way) with a mean score of 5.8 (SD = 0.89). The maximum scoring elements were element two (Engaging context for learning) and element six (How to improve or sustain good performance) with a mean score of seven.

## Discussion

This is a description of the development, implementation, and evaluation of a longitudinal SBL curriculum for PEM trainees. It utilized debriefing with good judgment and RCDP. Combining a more traditional curriculum development approach along with the instructional design ADDIE approach allowed us to thoughtfully and efficiently design a curriculum to meet the needs of the learners and address current training gaps in PEM training.

There is one other longitudinal SBL curriculum for PEM trainees in the literature [29]. There are a number of issues and differences worth noting. First, the curriculum described by Chen, et al. does not describe their development process with significant detail. Providing significant details about our instructional design and curriculum development process makes it more generalizable and easier to reproduce or adapt to other training program needs.

Second, PEM fellowship training programs in Canada are two years in length versus three years in the United States. The difference in training time can potentially make a difference when considering how to integrate SBL into PEM training programs.

Third, there is distinct difference in evaluation strategies between the two curriculums. Cheng et al. only used a 12-item satisfaction survey to evaluate their curriculum [29]. We implemented a robust evaluation strategy with numerous methods in an attempt to more completely evaluate learner and instructor performance as well as performance of the curriculum itself. By using a more robust evaluation strategy, we are well set-up to make changes to accommodate future learner needs.

Other published curriculums for this learner group are not as thorough as this curriculum. They tend to focus on either a single training gap such as trauma management [51] or delivering challenging news [52], focus on a single piece of the curriculum development process [53], or do not as thoroughly describe their process [51]. As a result, we believe our curriculum is the most generalizable and adaptable curriculum to date.

The 68% attendance is low but is as expected given the overall fellowship curricula setup. During curricular planning and development, a 66% attendance was anticipated. Given the 68% attendance rate, the 12-month curricular structure exposes the learners to the entire curriculum twice during their three years of fellowship training, potentially reinforcing and generalizing learning.

An important training gap identified during the needs assessment phase is a lack of clinical opportunities to perform critical procedures. It has been shown that PEM fellows perform a median of three critical procedures in a 12-month period [1]. This curriculum provided nine times that amount per year. Although learners only completed 67.5% of potential critical procedure opportunities, this does fall in-line with learner attendance (68%) implying procedure opportunities are evenly and well-spaced throughout the curriculum. What remains unclear, is if this increase in SBL procedural experience translates to clinical success with this learner group.

Instructor quality was also rated highly via DASH-SV© evaluations. The DASH-SV© is a well-established debriefing assessment tool with sound validity evidence [41, 50]. Utilizing this type of learner feedback is important in the context of the overall evaluation strategy for a few reasons. First, it provides direct learner feedback to the instructor in real-time. This allows instructors to adapt and alter their approaches as needed to better meet the needs of the learners. Second, it allows learners to potentially feel more involved with the curriculum development and adjustment process. Lastly, this type of evaluation allows for comparisons between instructors. This information can be used to maximize instructor quality longer term.

Overall, instructor performance was rated very highly. Element three of the DASH-SV©, structuring the debriefing in an organized way, scored the lowest of the seven elements. Effort will be put into improving element three.

There are several limitations to our study. First, the primary author was the assistant fellowship program director. This may have led to biased survey responses. This risk was minimized by the anonymous nature of the surveys. There is also potential bias from the end of curriculum in-person focus group as the primary author lead the focus group. Going forward, this risk will be minimized by having a person not connected to the curriculum or the fellowship lead the focus group. Second, the small number of study subjects makes assessment and generalization of results challenging. Related, is the lack of quantitative data regarding critical procedure performance. This was due to the current low *n* and would not provide meaningful information. This data collection is ongoing and at this time, being used for individual learner feedback. These limitations will be minimized going forward as more learners participate and more data is collected.

## Conclusion

This curriculum received a positive response from learners. Formative and summative assessments found increased critical procedure opportunities and high instructor performance. Future changes include more ultrasound integration, in-situ simulations, and a peer-to-peer education component. Continual assessment and sound instructional design processes will further revise curriculum going forward.

## Abbreviations

ABP: American Board of Pediatrics
ACGME: Accreditation Council for Graduate Medical Education
ADDIE: Analysis, Design, Development, Implementation, and Evaluation
CRM: Crisis resource management
EPA: Entrustable professional activities
IS: Instructional strategy (ies)
PEM: Pediatric Emergency Medicine
RDCP: Rapid cycle deliberate practice
SBL: Simulation-based learning
